# Progressive Conversion Model Applied to the Physical Activation of Activated Carbon from Palm Kernel Shells at the Pilot Scale in a Nichols Furnace and at the Industrial Scale in a Rotary Kiln

**DOI:** 10.3390/molecules30071573

**Published:** 2025-03-31

**Authors:** Ernesto de la Torre, Alex S. Redrovan, Carlos F. Aragón-Tobar

**Affiliations:** Department of Extractive Metallurgy, Escuela Politécnica Nacional, Ladrón de Guevara E11-253, P.O. Box 17-01-2759, Quito 170525, Ecuador; alex1998saul@hotmail.com (A.S.R.); carlos.aragont@epn.edu.ec (C.F.A.-T.)

**Keywords:** progressive conversion model, physical activation, activated carbon, pilot scale

## Abstract

Palm kernel shells, an abundant agro-industrial residue in countries like Ecuador, can be valorized through their conversion into activated carbon for industrial applications. This study investigates the physical activation of carbonized palm kernel shells using both a Nichols furnace at the pilot scale and a rotary kiln at the industrial scale. The progressive conversion model was used to explain how the activation process works and to calculate the reaction rate constants for CO_2_ (kr_CO2_) and H_2_O (kr_H2O_). The experimental results demonstrated that activation in an H_2_O-rich atmosphere significantly enhanced porosity development and iodine index compared to CO_2_ alone. Additionally, the study confirmed that activation kinetics are primarily controlled by the chemical reaction rather than mass transport limitations, as indicated by the negligible effect of particle size on gasification rates. At 850 °C, the reaction rate constants were calculated to be kr_CO2_ = 0.75 (mol·cm^−3^·s)^−1^ and kr_H2O_ = 8.91 (mol·cm^−3^·s)^−1^. The model’s predictions closely matched the experimental data, validating its applicability for process optimization at both the pilot and industrial scales. These findings provide valuable insights for improving the efficiency of activated carbon production from palm kernel shells in large-scale operations.

## 1. Introduction

The African palm kernel shell is an agro-industrial waste widely available in countries such as Ecuador [1,2,3]. The physicochemical characteristics of this waste make it a promising raw material to produce activated carbon [1,2]. Transforming these shells into activated carbon not only adds value to this waste but also generates a high-value product with multiple industrial applications. In the production process of activated carbon, physical activation is a crucial stage for developing the desired properties of the material [4,5]. Given the importance of this process, it is essential to study it not only at the laboratory scale but also at the pilot and industrial scales, where it is performed under more complex conditions [1,3,4].

The first step in this transformation involves the carbonization of palm kernel shells through pyrolysis. De la Torre and Gámez [6] describe this process using the Not Reacted Core Model. Subsequently, the carbonized material undergoes a physical activation stage using carbon dioxide (CO_2_) and water vapor (H_2_O) to enhance its porosity. According to Gómez et al. [7,8], the specific surface area developed during the carbonization of palm kernel shells is limited, reaching only 4 m^2^·g^−1^.

Additionally, Raveendran et al. [9] demonstrated that the pores formed during the pyrolysis of lignocellulosic materials are often blocked by tar deposits generated through condensation reactions. Physical activation removes these condensed tars and develops high internal porosity. This process, carried out in controlled-atmosphere furnaces (using H_2_O vapor and/or CO_2_), operates at temperatures ranging from 800 to 1000 °C [3,10,11]. During activation, the carbonized material undergoes decomposition through carbon gasification, forming CO and CO_2_. This process is accompanied by the release of volatile compounds rich in oxygen and hydrogen. The final product is activated carbon with an advanced porous structure [2,3,4,12]. 

Manocha [13] and Marsh and Reinoso [14] highlighted that carbon atoms exhibit varying reactivities depending on their structural environment. Thus, carbon atoms associated with functional groups are more reactive. Other highly reactive atoms are found in the defects of polycyclic aromatics (which form the base of benzene). Additionally, carbon atoms bonded to small amounts of heteroatoms, such as nitrogen (N), oxygen (O), and hydrogen (H), are also reactive.

During physical activation, reactions (1) and (2) serve as the basis for the gasification of carbon in carbonized materials. Alongside each reaction, the enthalpy values (ΔH°) and expressions for calculating Gibbs free energy (ΔG°) as a function of temperature T (expressed in K) are provided [1,11,14,15,16,17,18,19]:

C(s) + CO_2_ (g) = 2 CO (g) (1)

ΔH°_Rx.1_ = +172 kJ·mol^−1^ and ΔG°_Rx.1_ = +172 − 0.176 T (kJ·mol^−1^)

C (s) + H_2_O (g) = CO (g) + H_2_ (g) (2)

ΔH°_Rx.2_ = +131 kJ·mol^−1^ and ΔG°_Rx.2_ = +131 − 0.133 T (kJ·mol^−1^)

Both reactions are endothermic and occur spontaneously at 1 atm starting at 704 °C (reaction (1)) and 711 °C (reaction (2)). At 900 °C, a typical temperature for the activation process, Reaction 1 has a Gibbs free energy value of ΔG_Rx.1_ = −25.00 kJ·mol^−1^, whereas Reaction 2 has a Gibbs free energy value of ΔG_Rx.2_ = −34.44 kJ·mol^−1^. At this temperature, both reactions become fully spontaneous. However, due to their endothermic nature, precise control of furnace conditions is crucial to ensure efficient gasification and adequate pore development during activation [11,20].

Additionally, the reaction with CO_2_ requires 41 kJ·mol^−1^ more energy than the reactions with H_2_O. The H_2_O molecule, being smaller, diffuses more quickly into the carbon pores, making its activation more efficient [3,9,12,17,21]. Indeed, Kunii and Chisaki [22] demonstrated that, at 854 °C, the reaction of H_2_O vapor (reaction (2)) is ten times faster than the activation with CO_2_ (reaction (1)). The development of porosity in the carbonized material is directly related to the activation time and the percentage of carbon gasification, known as “burn-off” or degree of activation. The “χ”, or “conversion”, is determined by weighing the material before and after processing.

According to Marsh and Reinoso [14], CO_2_ or H_2_O molecules must contact carbon atoms 10^17^ times before achieving gasification. This number of interactions is comparable to the number of CO_2_ molecules present in the porosity of a carbonaceous material during gasification. Also, this number of interactions is comparable to the number of N_2_ molecules adsorbed in carbonaceous materials at −196 °C and a pressure of 0.1 MPa. Consequently, in the structure of carbonaceous materials, carbon atoms do not have the same reactivity, and as a result, porosity can develop inside the particles through the selective and preferential gasification of the most reactive carbon atoms.

Studies conducted by Manocha [13] on the activation of pine wood at 750 °C with CO_2_ and H_2_O vapor revealed that activation with CO_2_ generates a greater proportion of micropores, accounting for 90% of the specific surface area and 73% of the total pore volume. In contrast, activation with H_2_O vapor produces fewer micropores (63% of the specific surface area) and develops more mesopores, contributing only 33% of the total pore volume [11,23]. Daud and Ali [12] also distinguished between supermicropores, with diameters ranging from 0.7 to 2.0 nm, and ultramicropores, with diameters smaller than 0.7 nm.

Additionally, Marsh and Reinoso [14] analyzed activated carbons produced from olive stones activated with CO_2_ and H_2_O vapor at 800 °C. Their results indicated that CO_2_ favored the formation of micropores and ultramicropores (“narrow microporosity”), whereas H_2_O vapor promoted the development of mesopores. Furthermore, they confirmed a linear relationship between the carbon gasification rate and the activation time, noting that gasification rates are identical for CO_2_ and H_2_O [3,11].

Physical activation is a widely used method for producing activated carbon from palm kernel shells, relying on high temperatures (typically between 800 and 1000 °C) and an oxidizing gas, such as steam or carbon dioxide, to activate the material. This method tends to produce a higher proportion of micropores (pores smaller than 2 nanometers), resulting in activated carbon with a very high surface area and excellent adsorption properties for small molecules. Although physical activation requires intense heat and extended processing times, it is considered an environmentally friendly method, as it does not involve chemical agents.

On the other hand, chemical activation involves treating the palm kernel shells with a chemical agent, such as phosphoric acid or potassium hydroxide, at relatively lower temperatures (typically between 400 and 600 °C). This process primarily creates mesopores (pores with diameters between 2 and 50 nanometers) and macropores (pores larger than 50 nanometers), leading to a carbon structure that is highly porous and effective at adsorbing larger molecules. Activation with phosphoric acid is particularly effective for lignocellulosic materials, as it generates a well-developed porosity while maintaining high yields [24]. Similarly, potassium hydroxide activation produces highly porous carbon with superior adsorption characteristics compared to other chemical activators [25]. While chemical activation may yield a carbon material with a more refined pore structure for specific applications, physical activation remains preferred for its simplicity, reduced chemical usage, and ability to produce a broader range of pore sizes.

### Application of the Progressive Conversion Model to the Physical Activation

Kunni and Chisaki [22] consider that the physical activation process of carbon in the presence of CO_2_ and H_2_O vapor can be represented by the following general reaction:

A(g) + bB(s) => D(g) + E(s) (3)

where

A(g): gaseous reagent (CO_2_ and H_2_O);
B(s): carbonized material (solid) to be activated;
D(g): gas produced during activation;
E(s): activated carbon (solid) produced by activation.

The progressive conversion model that describes the activation is presented in Equation (4):

χ_B_ = 1 − exp(−k_r_ C_A_ t) (4)

where

χ_B_: conversion rate of carbon—gasification of carbon (%);
k_r_: specific reaction rate constant (mol·cm^−3^·s)^−1^;
C_A_: gas concentration (mol·cm^−3^);
t: time (s).

This model assumes that the carbon gasification reaction is solely controlled by reaction kinetics, without considering phenomena related to mass and heat transport.

The aim of this study is to understand the phenomena involved during the physical activation of palm kernel shells by CO_2_ and H_2_O vapor. This analysis is not only necessary at the laboratory scale but also, more importantly, at the pilot and industrial scales, where the activation process conditions are more complex and representative of real-world applications. In this study, the progressive conversion model is applied to predict the activation of carbonized material derived from palm kernel shells. The theoretical results obtained using this model are compared with experimental data collected during the activation of palm kernel shells using a mixture of CO_2_ and H_2_O vapor. Experimental tests were conducted in a Nichols furnace at the pilot scale and a rotary kiln at the industrial scale.

## 2. Results

### 2.1. Influence of Furnace Atmosphere on the Gasification Rate and Iodine Index

**Figure 1:**
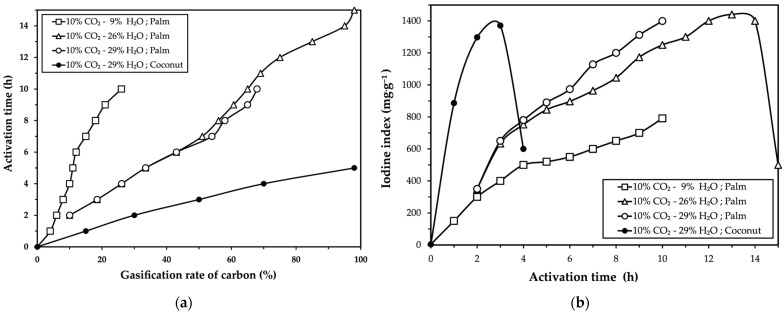
Effect of furnace atmosphere on the (**a**) carbon gasification rate and (**b**) iodine index of carbonized palm kernel shells [4 kg sample (−7 + 4 mm), 4 RPM, λ = 0.76, 960 °C], including comparative results for coconut shell activation.

The physical activation of the carbonized material produced from palm kernel shells was studied by analyzing the carbon gasification percentage and the iodine index. The evolution of these two parameters as a function of activation time is presented in Figure 1a,b, respectively. Additionally, the experimental results for the activation of carbonized coconut shells are included as a reference in the same figures.

Figure 1a shows a general increase in activation time with carbon gasification percentage, although the relationship appears to be curvilinear rather than strictly linear. In furnace atmospheres with a lower water content (9% H_2_O), the carbon gasification rate was slower compared to atmospheres with higher water contents (26% and 29% H_2_O). On the other hand, Figure 1b demonstrates that the iodine index of the activated carbon increases with activation time.

**Figure 2:**
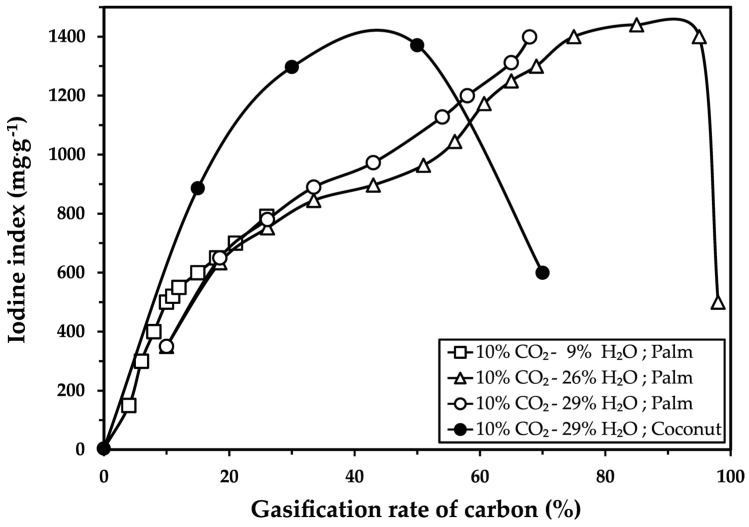
Iodine index as a function of the gasification rate of carbonized palm kernel shells under three different atmospheric conditions [4 kg sample (−7 + 4 mm), 4 RPM, λ = 0.76, 960 °C], including comparative results for coconut shell activation.

Figure 2 presents the iodine index as a function of the gasification rate of carbon for palm kernel shells in three different atmospheric conditions and compares these results to those of coconut shells.

As shown in Figure 2, the iodine index of the activated carbon obtained from palm kernel shells increased with the carbon gasification percentage, reaching a maximum value of 1400 mg·g^−1^ after 13 to 14 h of activation in a 10% CO_2_ and 26% H_2_O atmosphere, with 95% gasification. In contrast, the coconut shells reached their maximum iodine index of 1370 mg·g^−1^ after only 3 h of activation, with 50% gasification.

A maximum value in the iodine index was observed, followed by a decline in this value with longer activation times, as shown in Figure 1b and Figure 2. This trend is linked to the collapse of the carbon’s porous structure. In the case of coconut shell carbon, after 4 h and with a 70% gasification rate, the iodine index decreased by 56% from its maximum, reaching only 600 mg·g^−1^. For palm kernel shell carbon, after 15 h, the iodine index decreased by 65% from its maximum, reaching only 500 mg·g^−1^ with a 98% gasification rate.

### 2.2. Influence of Temperature on the Physical Activation of Palm Kernel Shells at the Pilot Scale

**Figure 3:**
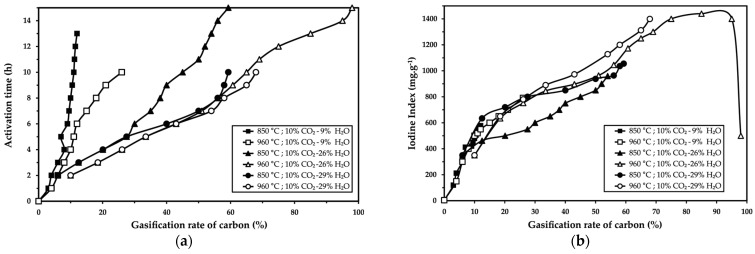
Influence of furnace temperature (850 °C and 960 °C) and atmosphere (10% CO_2_-9% H_2_O; 10% CO_2_-26% H_2_O and 10% CO_2_-29% H_2_O) on (**a**) the activation time and (**b**) the iodine index for carbonized palm kernel shells [4 kg of sample (−7 + 4 mm); 4 RPM^−1^; λ = 0.76].

The influence of two working temperatures (850 °C and 960 °C) during the physical activation of carbonized palm kernel shells was studied. The results are presented in Figure 3a, which shows the carbon gasification rate as a function of activation time, and in Figure 3b, which shows the iodine index as a function of activation time. Three different atmospheric conditions were evaluated: 10% CO_2_-9% H_2_O, 10% CO_2_-26% H_2_O, and 10% CO_2_-29% H_2_O.

Figure 3a shows that the higher temperature (960 °C) increased the gasification rate in a shorter time for all three atmospheric conditions. For example, in an atmosphere of 10% CO_2_-9% H_2_O, a gasification rate of approximately 25% was achieved after 10 h at 960 °C, whereas only 10% gasification was obtained after 13 h at 850 °C. Similarly, in an atmosphere of 10% CO_2_-26% H_2_O, a gasification rate of 95% was achieved after 15 h at 960 °C, compared to only 60% gasification at 850 °C in the same timeframe. Finally, for an atmosphere of 10% CO_2_-29% H_2_O, a gasification rate of 65% was observed after 10 h at 960 °C, while only 55% gasification was achieved at 850 °C.

The iodine index showed similar trends. For an atmosphere of 10% CO_2_-9% H_2_O, the maximum iodine index values were 578 mg·g^−1^ at 850 °C and 791 mg·g^−1^ at 960 °C. In an atmosphere of 10% CO_2_-26% H_2_O, the maximum iodine index reached 960 mg·g^−1^ at 850 °C and 1440 mg·g^−1^ at 960 °C. Finally, for an atmosphere of 10% CO_2_-29% H_2_O, the maximum iodine index values were 1055 mg·g^−1^ at 850 °C and 1399 mg·g^−1^ at 960 °C.

### 2.3. Influence of Particle Size on Gasification Kinetics

**Figure 4:**
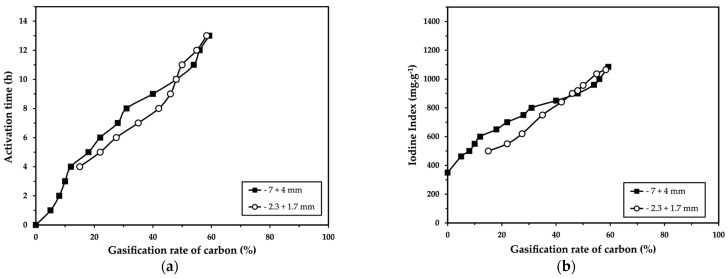
Influence of particle size (−7 + 4 mm and −2.3 + 1.7 mm) on (**a**) activation time and (**b**) iodine index as function of gasification rate of palm kernel shells during physical activation in the presence of 10% CO_2_- – 26% H_2_O [4 kg of sample; 4 RPM^−1^; λ = 0.76; 850 °C].

The influence of particle size on the gasification kinetics (10% CO_2_-26% H_2_O) of palm kernel shells and on the iodine index is presented in Figure 4.

For the two particle size ranges tested (−7 + 4 mm and −2.3 + 1.7 mm), no significant influence of particle size was observed on the gasification kinetics or the iodine index.

### 2.4. Application of the Progressive Conversion Model in the Physical Activation of Palm Kernel Shells

Table 1 summarizes the results obtained after applying the progressive conversion model to determine the specific rate of reaction for physical activation by CO_2_ and H_2_O for carbonized palm kernel shells.

As shown in Table 1, increasing the activation temperature from 850 °C to 960 °C results in the specific reaction rate constant for CO_2_ (kr_CO2_) doubling, while the specific reaction rate constant for H_2_O (kr_H2O_) increases by a factor of 1.3. Additionally, kr_H2O_ is approximately five times higher than kr_CO2_ at 850 °C, but this ratio decreases to three times at 960 °C.

**Figure 5:**
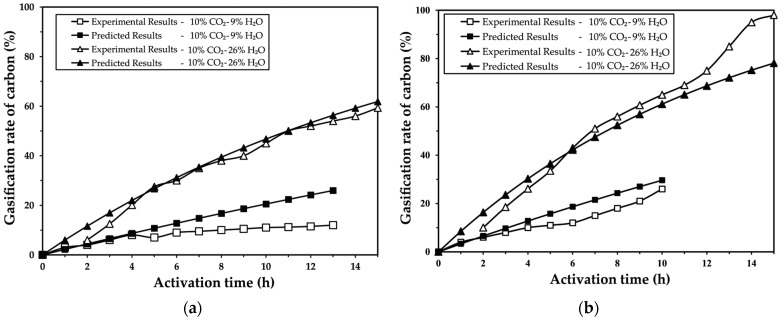
Gasification rate of carbon calculated by applying the progressive conversion model compared to experimental results obtained in the Nichols furnace during physical activation at (**a**) 850 °C and (**b**) 960 °C for carbonized palm kernel shells [4 kg of sample (−7 + 4 mm); 4 RPM-1; λ = 0.76].

Figure 5 presents the comparison between the experimental results and the predicted results using the progressive conversion model for the physical activation of palm kernel shells.

Figure 5 illustrates that, when using the specific reaction rate constants calculated from the tests conducted in the Nichols furnace, presented in Table 1, the continuous conversion model fits very well with the experimental results of the physical activation of carbon derived from palm kernel shells.

For the physical activation of carbonized palm kernel shells with a particle size of (−7 + 4 mm) at 850 °C and 960 °C in an atmosphere of 10% CO_2_-26% H_2_O, the iodine index (ψ) and the carbon conversion rate (χ) are related, respectively, by Equation (5) (temperature = 850 °C) and Equation (6) (temperature = 960 °C). For simplicity and practical purposes, the linear approximation was chosen because it provides a good representation of the data within the range analyzed, with an R² value greater than 0.95, indicating a strong fit between the iodine index and the conversion rate of carbon.

ψ = 12.96 χ + 238.86 (5)

ψ = 15.87 χ + 301.36(6)

where

ψ: iodine index (mg·g^−1^);
χ: conversion rate of carbon (%).

### 2.5. Production of Activated Carbon from Palm Kernel Shells in an Industrial Rotary Kiln

**Figure 6:**
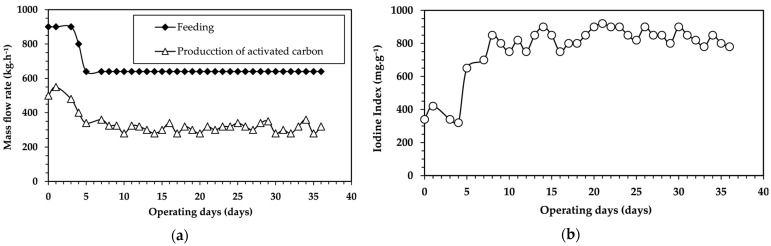
(**a**) Mass flow rate and (**b**) iodine index for the physical activation of carbon from palm kernel shells (−7 + 4 mm) in an industrial rotary kiln with lifters (average feed: 600 kg·h^−1^, temperature: 850 °C, kiln rotation speed: 0.3 rpm^−1^, average residence time: 5.4 h).

The results of the physical activation tests, in terms of mass flow rate and iodine index, using the rotary kiln with lifters are presented in Figure 6.

As presented in Figure 6a, an initial feed rate of 900 kg·h^−1^ produced an activated carbon with an iodine index of only 300 mg·g^−1^. When the feed rate was reduced to 600 kg·h^−1^, the activated carbon flow rate was 314 ± 62 kg·h^−1^, with an iodine index of 800 ± 162 mg·g^−1^ and a carbon gasification rate of 50%. The presence of lifters inside the kiln enhanced the contact between solids and gases, thereby improving the activation process.

The observed variability in the iodine index values and the activated carbon output rate can be attributed to fluctuations in the volatile material content of the palm kernel shells, which alter the furnace atmosphere. Additionally, the presence of oxygen in the kiln may oxidize the carbon, limiting pore formation and thus reducing porosity development in the activated material.

## 3. Discussion

### 3.1. Impact of Furnace Atmospheric Composition on Gasification and Porosity Development During Physical Activation

The results presented in Figure 1 align with similar trends observed in the activation of palm kernel shells and coconut shells with CO_2_, as reported by Daud and Ali [12] and Niu et al. [26], in the activation of coconut shells with H_2_O vapor described by McDougall [27], and in the activation of palm kernel shells with N_2_-H_2_O reported by Gómez et al. [7,8].

Figure 1a highlights that the presence of H_2_O vapor in the furnace atmosphere enhances the carbon gasification rate. This behavior is attributed to the molecular properties of H_2_O, as its smaller molecule size compared to CO_2_ allows for faster diffusion into the pores of the carbonized material, thereby increasing carbon reactivity and promoting porosity development. These observations are consistent with findings from Manocha [13], Marsh and Reinoso [14] and McDougall and Hancock [18].

Meanwhile, the results shown in Figure 2 indicate that the higher gasification rate observed in palm kernel shells (95%) makes it more challenging to develop a porous structure in the carbonized material. In contrast, coconut shells, with their lower cellulose and hemicellulose contents, exhibit destruction of their porous structure only after reaching 50% gasification. These differences reflect the distinct structural and chemical properties of the two precursors. Specifically, the carbon atoms in coconut shells are more reactive due to their less compact molecular structure, as described by Marsh and Reinoso [14], Shabir et al. [15], Tsai et al. [16] and Vi et al. [17].

Marsh and Reinoso [14] also emphasize that the structural environment of carbon atoms varies significantly within carbonaceous materials. The location of carbon atoms directly influences their susceptibility to react with gases like CO_2_ and H_2_O during activation. For instance, these gases tend to preferentially react with carbon atoms in graphene sheets. Additionally, the higher reactivity of certain carbon atoms is associated with unbalanced valences, which increases their interaction with activating gases.

Finally, these results agree with those obtained for the activation of soy charcoal with H_2_O vapor at 0.5 atm, as reported by Kunii and Chisaki [22], and for the activation of coconut shells described by McDougall and Hancock [18]. However, they contradict the findings of Marsh and Reinoso [14] and Rodríguez-Reinoso et al. [21] regarding the activation of olive stones with CO_2_ and H_2_O, where identical carbon gasification rates were reported for both gases.

The performance of activated carbon production is closely related to the composition of the biopolymers that make up the lignocellulosic raw materials from which the material is derived. Therefore, making a comparison with literature data is not entirely conclusive. However, in the study by Cagnon et al. [28], 10 g of coconut shell produced 3.36 g of carbonized material, and with the use of activation, 2 g of activated carbon was finally produced (22% gasification). In the case of this study, a 30% yield was achieved for carbonization, and 50% of gasification was attained for activated carbon production from palm kernel shells. In general, from 10 g of palm kernel shell, an approximate production of 1 g of activated carbon was achieved. While the data from this study are consistent with those presented in the literature, they should be considered only as a reference, given the specificity of the raw material and the carbonization–activation method used in the different furnaces.

### 3.2. Effect of Temperature on Gasification Kinetics and Porosity Development

The results demonstrate that an increase in temperature enhances both the gasification kinetics and the development of porosity. This is evident from the differences observed in gasification rates and iodine index values at 850 °C and 960 °C for the three atmospheric conditions evaluated.

The increase in gasification rate observed at 960 °C for all three atmospheres can be attributed to the accelerating effect of temperature on the chemical reactions involved in the activation process. Higher thermal energy facilitates the breaking of carbon bonds and promotes interactions with activating gases (CO_2_ and H_2_O), resulting in a more efficient carbon conversion.

The iodine index, which reflects the development of porosity, also exhibits a clear dependence on temperature. For all the atmospheres tested, the maximum iodine index values at 960 °C were significantly higher than those at 850 °C. This indicates that the higher temperature not only accelerates gasification kinetics but also promotes greater pore opening and porosity development in the carbonized material.

For the case of 10% CO_2_-26% H_2_O, shown in Figure 3, the iodine index reached a maximum value of 1440 mg·g^−1^ at 960 °C, compared to 960 mg·g^−1^ at 850 °C. This highlights the role of water vapor as an activating agent, as its smaller molecular size allows for greater diffusion into the pores of the carbonized material, facilitating the development of a more complex porous structure.

These findings are consistent with previous studies, such as those reported by Marsh and Reinoso [14] and McDougall and Hancock [18], who also observed that higher temperatures and the presence of water vapor significantly increase carbon reactivity and porosity generation.

### 3.3. Role of Particle Size in Physical Activation

The results suggest that particle size does not play a significant role in gasification kinetics or the iodine index within the tested size ranges. This supports the hypothesis that activation by CO_2_ and H_2_O steam is primarily controlled by the kinetics of the chemical reaction, rather than by mass transport-related factors.

### 3.4. Analysis of the Progressive Conversion Model and Its Application at the Industrial Scale

The results from this study demonstrate that increasing the activation temperature significantly influences the specific reaction rate constants for CO_2_ and H_2_O. Specifically, kr_H2O_ is consistently higher than kr_CO2_, confirming the critical role of water vapor as an activating agent. However, as the temperature increases from 850 °C to 960 °C, the kr_H2O_/kr_CO2_ ratio decreases from 5 to 3, indicating that the relative contribution of CO_2_ to the gasification process becomes more significant at higher temperatures.

The specific reaction rate for H_2_O at 850 °C reported by Kunii and Chisaki [22] was 46 (mol·cm^3^·s)^−1^ during the activation of 6.4 mm coconut shells under an atmosphere of 50% H_2_O and 50% N_2_. This value is comparable to the results obtained in this study for the activation of carbonized palm kernel shells. This finding supports the applicability of the continuous conversion model to different carbonaceous materials, including palm kernel shells.

Additionally, as shown in Figure 5, the continuous conversion model aligns closely with the experimental data, reinforcing the hypothesis that chemical reaction kinetics is the dominant factor controlling activation under the evaluated conditions.

Finally, the consistency of the specific reaction rate constants (kr_CO2_ and kr_H2O_) obtained at the pilot scale enables their use in calculating the variation of the iodine index as a function of the length of an industrial rotary kiln with lifters. In such cases, due to the absence of significant limitations related to heat and mass transport, the residence time required for a specific conversion at the pilot scale can be directly applied at the industrial scale.

Since the activation model obtained was accurate for the furnace at the pilot level, these results were also used for activation at the industrial level. However, although the model can be extrapolated, it is important to highlight some differences between the two furnaces studied. On the one hand, the Nichols furnace is a pilot-scale furnace that operates with a flame produced by the combustion of liquefied petroleum gas (LPG) and, due to its design characteristics, allows for precise control over the composition of the gases in the atmosphere. The control of these gases enabled the evaluation of the kinetics of the reactions occurring inside the furnace. This feature of the pilot-scale furnace was advantageous for applying the progressive conversion model.

However, the direct application of the progressive conversion model at the industrial scale presents more challenges. For instance, the industrial rotary furnace, which uses diesel as fuel, operates in an oxidizing atmosphere with the intake of parasitic air, which burns part of the activated carbon and reduces efficiency, making it more difficult to control the atmosphere compared to the pilot-scale furnace. In contrast, the Nichols furnace allows for more precise and reliable determination of kinetic parameters. Considering these limitations in controlling parameters in the rotary kiln, the model developed for the Nichols furnace was used to predict carbon behavior during physical activation.

### 3.5. Validation and Challenges of Applying the Progressive Conversion Model at an Industrial Scale

The continuous conversion model developed and validated in the previous section for the Nichols furnace can be applied to describe the physical activation in the rotary kiln. The model developed has been used to obtain the evolution of the iodine index within the kiln as a function of kiln length. Determining this variation in an industrial kiln is quite challenging, as the primary focus is the obtention of activated carbon as the final product. In normal operation, only the final product is possible to characterize. Characterization of the intermediate product inside the kiln is not possible from sampling during operation. In fact, taking samples at different points along the kiln is impractical. For this reason, the model developed in this study offers a useful approach for understanding the evolution of the iodine index along the length of the industrial kiln without the need to collect data during the kiln operation.

**Figure 7:**
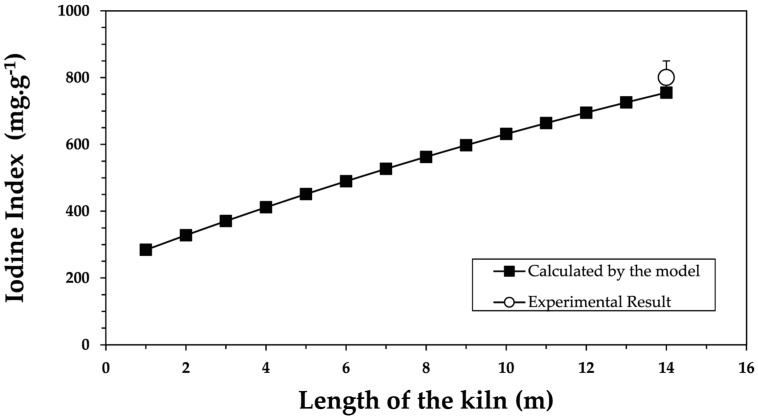
Comparison of the iodine index values calculated by the application of the “continuous conversion” model and the experimental values of the physical activation of the carbonized shells in the industrial rotary kiln with lifters [600 kg·h^−1^ carbonized shells (−7 + 4 mm); 850 °C; 0.3 rpm^−1^; λ = 1.2; average residence time of 8.1 h; χ = 0.38%].

The residence time as a function of furnace length was calculated using Equation (8). The values of kr_CO2_ and kr_H2O_ for the activation in the rotary kiln were obtained from Table 1. These values were used to obtain the corresponding iodine index in the rotary kiln by applying Equation (7). Figure 7 compares the continuous conversion model with the experimental results.

Figure 7 shows that the continuous conversion model aligns closely with the experimental result at the exit from the kiln. In the Nichols furnace, operating at 850 °C with an atmosphere of 10% CO_2_-26% H_2_O, a carbon gasification rate of 45% corresponds to an iodine index of 800 mg·g^−1^, achieved with a residence time of 9 h. These values are comparable to those obtained in the industrial rotary kiln with lifters, where residence times of 8 to 5 h, depending on the rotation speed (0.2–0.3 rpm), resulted in similar iodine index values.

Consequently, the model developed and validated on the pilot scale was adapted for the industrial scale with promising perspectives, particularly under conditions with minimal mass and heat transfer limitations. The evolution in iodine index values along the length of the kiln, as predicted by the model, provides valuable insights. This finding can serve as a crucial starting point for gaining a deeper understanding of the physical activation process inside the kiln, particularly in cases where experimental data are scarce or difficult to obtain.

## 4. Materials and Methods

### 4.1. Physical Activation of Carbonized Palm Kernel Shells and Coconut Shells in a Nichols Furnace

The palm kernel shells (*Tenera* variety) were obtained from an oil palm producer located in the Province of Esmeraldas, Northwest Ecuador. Coconut shells were collected from a local market in Ecuador. The collected material was cleaned thoroughly to remove any impurities.

The first step in the production of activated carbon from these residues was carbonization. The carbonized material was produced using either palm kernel shells or coconut shells in a Nichols furnace. This furnace consists of two interconnected chambers, each 457 mm in diameter per chamber (one designated for combustion and the other for reaction). The reaction chamber is equipped with an agitator rotating at four revolutions per minute (RPM). The Nichols furnace atmosphere was controlled using the lambda factor (λ), defined as the ratio of the air supplied to the burner to the stoichiometric air required for complete combustion of the fuel (a propane–butane mixture, 50% each). Air was supplied via a fan with a flow rate of 1.66 Nm^3^·min^−1^. The combustion gas flow rate was estimated at atmospheric pressure (0.72 atm). The composition of the furnace atmosphere was verified using a Testo 350 gas analyzer.

Carbonization was performed under a reducing atmosphere [λ = 0.76, characterized by flue gas composition: 10% CO_2_, 4% CO, 11% H_2_, 0.01% O_2_, and 9% humidity] in an agitated bed (4 RPM). An initial load of 4 kg of palm kernel shells or coconut shells, with particle sizes ranging between 5 and 20 mm, was processed for 3 h at the temperature specified for the activation test (850 and 960 °C). Details of the carbonized material from palm kernel shells can be found in De la Torre and Gámez [6]. After carbonization, the samples were crushed and sieved to obtain two fractions: −7 mm + 4 mm and −2.3 mm + 1.7 mm.

The physical activation process was conducted following methodologies proposed by Prauchner and Rodríguez-Reinoso [19] and Oudenne [29]. Activation was performed in the same Nichols furnace, using an agitated bed (4 RPM) containing a load of 4 kg of crushed and sieved carbonized material (palm kernel shells or coconut shells) under a reducing atmosphere (λ = 0.76) with a gas composition of 10% CO_2_, 4% CO, 11% H_2_, 0.01% O_2_, and 9% humidity. Saturated water vapor was produced using a 49 KW (5 BHP) electric boiler.

During the activation tests, the furnace atmosphere was controlled and maintained under three different condition, 10% CO_2_ and 9% H_2_O, 10% CO_2_ and 26% H_2_O, and 10% CO_2_ and 29% H_2_O, at two different temperatures (850 °C and 960 °C).

For each test, the furnace was preheated to the desired temperature and the samples were introduced only when the furnace reached this temperature. At the end of the test, the material was unloaded, cooled in a closed metal container, and weighed. The weight difference before and after activation was used to calculate the percentage of gasification. Samples were collected every hour until 10 to 14 h (for palm kernel shells) or 4 h (for coconut shells). These samples were analyzed to determine the iodine index as a measure of activation.

### 4.2. Single-Step Carbonization and Physical Activation of Palm Kernel Shells in a Continuous Rotary Kiln at an Industrial Level

The industrial-scale tests were conducted in a rotary kiln located in the province of Esmeraldas, northwest Ecuador. This kiln operates using fuel oil under an atmosphere with 20% excess air (λ = 1.2) and a burner thermal capacity of 8 × 10⁶ kJ·h^−1^. The raw material is fed into the kiln through a screw conveyor, moving in the same direction as the combustion gases (co-current flow). The treated material is discharged at the opposite end of the kiln via a water-cooled auger.

The combustion gases exit from the top of the kiln are directed to a post-combustion chamber, followed by a plate heat exchanger, a cyclone, an induced draft fan, and finally a chimney. The nominal solid feed capacity of the kiln is 1 m^3^·h^−1^. Depending on the rotation speed and the use of lifters, the material residence time varies between 0.3 and 8.0 h.

The rotary kiln features an automatic control system that regulates the flow of solid materials entering and exiting the kiln, fuel oil consumption, the temperatures of combustion gases at both the inlet and outlet, the temperature of gases at the inlet of the induced draft fan, the kiln rotation speed, and the consumption of cooling water. Additionally, the combustion gas circuit includes a 1.471 kW (or 150 BHP) boiler, which generates 2400 kg·h^−1^ of steam at a pressure of 5.4 atm, and a 250 kVA power generator.

Single-step carbonization and activation tests were conducted at an industrial scale using untreated palm kernel shell samples with particle sizes ranging from 4 to 7 mm. The rotary kiln was preheated for 7 days until it reached a temperature of 850 °C under an oxidizing atmosphere (λ = 1.2). Fuel oil consumption was 2650 L·day^−1^, equivalent to 1.47 L of fuel oil per kilogram of product.

The kiln rotation speed was set at three revolutions per minute, with an initial material feed rate of 900 kg·h^−1^. The production rate of carbonized materials and their volatile matter content were periodically measured. Each test lasted 35 days to ensure sufficient material production to meet potential customer demands. The activation atmosphere was generated by injecting water vapor at 1.4 atm into the combustion chamber. During these tests, the flue gas composition was 25% H_2_O, 10% CO_2_, and 5% O_2_. The flow rate of the activated carbon produced was measured. The characteristics of the carbon produced were evaluated using the iodine index.

### 4.3. Iodine Index Determination for Activated Carbon Characterization

The material recovered from the activation tests (1–3 g) at different times was initially pulverized (~74 µm). The pulverized material was mixed with 100 mL of an iodine (I_2_) solution at 0.1N for 30 seconds and then filtered. The adsorbed iodine value was determined by titrating the residual iodine in the solution using sodium thiosulfate (0.1 N) and starch (1% w·w^−1^) as an indicator. The iodine index was expressed as milligrams of I_2_ per gram of activated carbon. This determination was based on the standards ASTM D4607-86 (ASTM, 1986) [30] and AWWA B 600-78 (AWWA, 1978) [31].

### 4.4. Continuous Conversion Model Applied for Physical Activation

As a simplification for activation with CO_2_ and H_2_O, it was assumed that the overall conversion could be represented as the sum of individual conversions (presented in Equation (4)), which can be calculated using the following equation:

(7) $$\chi_{B}=\left[1-\exp\left(-k_{r\;{CO}_{2}\;}\;C_{A\;{CO}_{2}\;}t\right)\right]+[1-exp(-k_{r\;H_{2}O\;}\;C_{A\;H_{2}O\;}t)]$$

where

χ_B_: carbon conversion—carbon gasification by CO_2_ and H_2_O (%);
kr_CO2_: specific rate constant of reaction by CO_2_ (mol·cm^−3^·s)^−1^;
C_A CO2_: CO_2_ concentration (mol·cm^−3^);
t: time (s);
kr_H2O_: specific rate constant of reaction by H_2_O (mol·cm^−3^·s)^−1^;
C_A H2O_: H_2_O concentration (mol·cm^−3^).

The specific reaction rate constant (kr) for CO_2_ and H_2_O was calculated using the finite element method. The specific reaction rate constant for H_2_O vapor (kr_H2O_) was estimated under the assumption that the carbonized material conversion attributed to H_2_O vapor (χ_H2O_) corresponds to the difference between the conversion obtained in an atmosphere of 10% CO_2_ and 26% H_2_O (χ_10% CO2-26% H2O_) and the conversion obtained in an atmosphere of 10% CO_2_ and 9% H_2_O (χ_10% CO2-9% H2O_).

Similarly, the specific reaction rate constant for CO_2_ (kr_CO2_) was estimated under the assumption that the carbon conversion due to CO_2_ (χ_CO2_) corresponds to the difference between the conversion obtained in an atmosphere of 10% CO_2_ and 9% H_2_O (χ_10% CO2-9% H2O_) and the conversion caused solely by 9% H_2_O vapor (χ_9% H2O_). The latter value was determined previously using the kr_H2O_ constant. Detailed calculations of these estimations are available in the Appendix A.

### 4.5. Residence Time in a Rotary Kiln

Kunni et al. [22] and Perry et al. [32] proposed different methods for calculating the residence time in a rotary kiln, a critical parameter for ensuring the quality control of the carbonized product. Among these methods, Equation (6) is the most widely used. This empirical relationship estimates the residence time of a solid material in a rotary kiln as a function of its rotation speed:

(8) $$t_{r}=\frac{1.77\;L\;\sqrt{\theta }\;F}{P\;D\;n}$$

where

t_r_: residence time (min);
L: length of the kiln (14 m);
θ: angle of repose of the palm nut shells (34.7°);
F: characteristic factor of the interior of the rotary kiln, which is 2 for a kiln with lifters;
P: kiln slope (2°);
D: inner diameter of the kiln (1.5 m);
n: rotation speed of the kiln (revolutions per min).

## 5. Conclusions

The composition of the furnace atmosphere has a significant impact on the physical activation of carbonized palm kernel shells. The results demonstrated a correlation between activation time, carbon gasification rate, and iodine index, indicating that the kinetics of the process are controlled by the chemical reaction. Additionally, the presence of H_2_O vapor in the furnace atmosphere promoted faster porosity development and a higher iodine index compared to an atmosphere containing only CO_2_. This effect is attributed to the smaller molecular size of H_2_O compared to CO_2_, allowing H_2_O to diffuse more rapidly into the carbon pores and requiring less energy to react (41 kJ·mol^−1^ less), thereby increasing the gasification rate.

After 14 h of activation in an atmosphere of 10% CO_2_ and 26% H_2_O, 90% carbon gasification was achieved, and the activated carbon had an iodine index of 1400 mg·g^−1^. However, increasing the H_2_O content from 26% to 29% resulted in minimal differences, indicating that once the H_2_O concentration threshold is reached, further increases have a limited effect. These findings confirm that H_2_O vapor is the primary activating agent for optimizing carbon gasification and porosity development, as further validated by the reaction rate constants calculated at the pilot scale (kr_CO2_ = 0.75 mol·cm^−3^·s^−1^ and kr_H2O_ = 8.91 mol·cm^−3^·s^−1^ at 850 °C).

The experiments conducted with particle size ranges of (−7 + 4 mm) and (−2.3 + 1.7 mm) showed that particle size has a minimal impact on gasification kinetics and porosity development. The reaction rate constants for CO_2_ and H_2_O did not vary significantly between the particle sizes studied, suggesting that the process is primarily governed by the kinetics of the chemical reaction rather than by mass transport phenomena. This finding allows for greater flexibility in material preparation without compromising the quality of the activated carbon produced.

The progressive conversion model applied to the physical activation proved effective in describing the relationship between reaction rate constants, carbon gasification, and iodine index. The experimental data obtained at the pilot scale aligned closely with the model, confirming that activation under the evaluated conditions is governed by the kinetics of the chemical reaction.

The validation of the progressive conversion model at the pilot scale enabled the extrapolation of results to the industrial rotary kiln, demonstrating that, in the absence of significant mass and heat transport limitations, the residence time required for a specific conversion at the pilot scale is equivalent to that required at the industrial scale.

The progressive conversion model applied for the rotary kiln offered a valuable tool for understanding the evolution of the iodine index along the length of an industrial kiln, a task that is otherwise challenging due to the impracticality of sampling intermediate products during normal operation. This model serves as a key starting point for further investigation into the physical activation process at an industrial level, particularly when experimental data are limited or difficult to obtain.

The findings in this study underscore the importance of H_2_O as the dominant activating agent and validate the progressive conversion model as a predictive tool for optimizing the design and operation of the physical activation process, both at the pilot and industrial scales.

While this study focused on the application of the progressive conversion model to describe the gasification of carbon produced from palm kernel shells, several promising applications for this material can be identified. The produced activated carbon has a wide range of potential uses due to its favorable characteristics. For example, it has been successfully used for gold adsorption in the mining industry, the treatment of cyanide effluents, and the purification of solutions in industrial processes. Additionally, it has shown promise in wastewater treatment. Furthermore, the gasification reaction with CO_2_ could be harnessed for synthesis gas production, which could be used in metal reduction processes like iron production. These applications highlight the versatility of palm kernel shell-derived activated carbon. Although further experimental tests are necessary to evaluate these prospects in more detail, the progressive conversion model serves as a useful tool to characterize this material and guide its potential industrial-scale utilization.

## Figures and Tables

**Table 1:** Specific rate of reaction for physical activation by CO_2_ and H_2_O calculated using pilot tests in a Nichols furnace for carbonized palm kernel shells.

Rate Constant	Temperature (°C)	
	850	960
kr_CO2_ (mol·cm^−3^·s)^−1^	1.87	4.44
kr_H2O_ (mol·cm^−3^·s)^−1^	9.91	12.55

**Figure A1:**
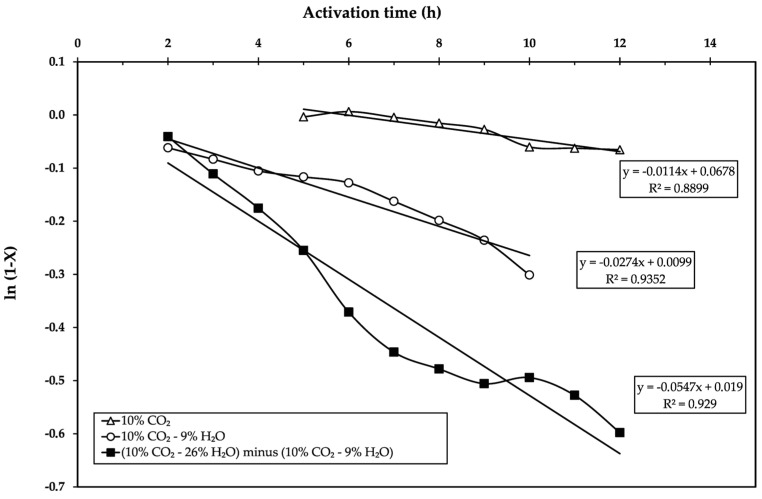
Graphical representation of equation A4 with the results of the physical activation of carbonized palm kelner shells in a Nichols furnace at 960°C.

## Data Availability

The original contributions presented in this study are included in the article. Further inquiries can be directed to the corresponding author.

## Appendix A

### Appendix A.1. Progressive Conversion Model for Physical Activation of Carbon Produced from Palm Kernel Shells

Calculation of the specific reaction rate constant for H_2_O (kr_H2O_) in the case of the physical activation test in the Nichols furnace at 960 °C was carried out as follows.

The estimation of the specific reaction rate constant for H_2_O (kr_H2O_) can be carried out using the results of pilot tests of the physical activation of carbonized palm kernel shells in the Nichols furnace at 960 °C and Equation (7) considering the hypothesis that the conversion of carbon due to H_2_O vapor (χ_H2O_) corresponds to the difference between conversion to an atmosphere of 10% CO_2_-26% H_2_O (χ_10% CO2 - 26% H2O_) and that of 10% CO_2_-9% H_2_O (χ_10% CO2-9% H2O_).

(A1) $$\chi_{H_{2}O}=\left(\chi_{{10\%\;CO_{2}-26\%\;H}_{2}O}\right)-\left(\chi_{{10\%\;CO_{2}-9\%\;H}_{2}O}\right)$$

(A2) $$\chi_{H_{2}O}=\left\{\left[1-\exp\left(-k_{r\;{CO}_{2}\;}\;C_{A\;{10\%\;CO}_{2}\;}t\right)\right]+[1-exp(k_{r\;H_{2}O\;}\;C_{A\;26\%\;H_{2}O\;}t)]\right\}-\left\{\left[1-\exp\left(-k_{r\;{CO}_{2}\;}\;C_{A\;{10\%\;CO}_{2}\;}t\right)\right]+[1-exp(k_{r\;H_{2}O\;}\;C_{A\;{9\%\;H}_{2}O\;}t)]\right\}$$

(A3) $$\chi_{H_{2}O}=-exp({-k}_{r\;H_{2}O\;}\;C_{A\;26\%\;H_{2}O\;}\;t)+exp({-k}_{r\;H_{2}O\;}\;C_{A\;{9\%\;H}_{2}O\;}t)$$

where

χ_H2O_: carbon conversion—carbon gasification by H_2_O (%);
kr_CO2_: specific reaction rate constant for CO_2_ (mol·cm^−3^·s)^−1^;
C_ACO2_: CO_2_ concentration (mol·cm^−3^);
t: time (s);
kr_H2O_: specific reaction rate constant for H_2_O (mol·cm^−3^·s)^−1^;
C_AH2O_: H_2_O concentration (mol·cm^−3^).

Equation (A3) can be approximated by Equation (A4), which is a straight line; with the value of its slope, it is possible to calculate kr_H2O_:

(A4) $$\ln\left(1-\chi_{H_{2}O}\right)={kr}_{H_{2}O}\;\left(C_{A\;26\%{\;H}_{2}o}-C_{A\;9\%{\;H}_{2}o}\right)x\;t$$

The estimation of the specific reaction rate constant for CO_2_ (kr_CO2_) can be calculated using Equation (7) considering the hypothesis that the conversion of carbonized palm kernel shells due to CO_2_ (χ_CO2_) corresponds to the difference between the conversion in an atmosphere of 10% CO_2_-9% H_2_O (χ_10% CO2-9% H2O_) and an atmosphere of 9% H_2_O vapor (χ_9% H2O_), which is determined by calculation with the kr_H2O_ obtained above.

The results of these calculations are presented in Table A1.

**Table A1:** Results of calculations of the gasification rate of carbon in different atmospheres based on the results obtained in pilot tests of the physical activation of palm kernel shells in the Nichols furnace at 960 °C.

Time (h)	Gasification Rate of Carbon—χ (%)		
	10% CO_2_-26% H_2_O minus 10% CO_2_-9% H_2_O	10% CO_2_-9% H_2_O	10% CO_2_
0	0.0	0.0	0.0
2	1.0	7.0	4.8
3	4.0	6.0	1.6
4	10.5	8.0	1.5
5	16.1	10.0	1.4
6	22.5	11.0	0.4
7	31.0	12.0	-0.6
8	36.0	15.0	0.4
9	38.0	18.0	1.5
10	39.7	21.0	2.7
11	39.0	26.0	5.9
12	41.0	28.0	6.1

Figure A1 presents the gasification rate of carbon calculated by applying the progressive conversion model compared to experimental results.

Figure A1 shows that the slope of the line corresponding to an atmosphere of 10% CO_2_-26% H_2_O minus 10% CO_2_-9% H_2_O is 0.0547 h^−1^.

$$C_{A\;26\%\;H_{2}O}-C_{A\;9\%\;H_{2}O\;}=\frac{P\times V_{H2O}}{R\times T\times Vt}$$

$$C_{A\;26\%\;H_{2}O}-C_{A\;9\%\;H_{2}O\;}=\frac{0.72\;atm\times \left(26-9\right)\times 10^{-3}L}{0.082\;\frac{L\;atm}{K\;mol}\times \left(96+273\right)K\times 100\;{cm}^{3}}$$

$$C_{A\;26\%\;H_{2}O}-C_{A\;9\%\;H_{2}O\;}=1.2106\times 10^{-6}\;mol\;{cm}^{-3}$$

In Equation (A4):

$${kr}_{H_{2}O}=\frac{slope\;[\left(10\%CO_{2}-26\%H_{2}O\right)-\left(10\%CO_{2}-9\%H_{2}O\right)]}{C_{A26\%H_{2}O}-C_{A9\%H_{2}O}}$$

$${kr}_{H_{2}O}=\frac{0.0547\;{cm}^{3}}{1.2106\times 10^{-6}\times 3600\;mols}$$

$${kr}_{H_{2}O}=12.55\;{cm}^{3}{mol}^{-1}s^{-1}$$

Figure A1 also highlights that the slope for the line corresponding to a 10% CO_2_ atmosphere is 0.0114 h^−1^:

$$C_{A10\%{\mathrm{CO}}_{2}}=\frac{P\times V_{{CO}_{2}}}{R\times T\times Vt}$$

$$C_{A10\%{\mathrm{CO}}_{2}}=\frac{0.072\;atm\times 10\;x\;10^{-3}L}{0.082\;\frac{L\;atm}{K\;mol}\times \left(960+273\right)\;K\times 100\;{cm}^{3}}$$

C_A 10% CO2_ = 7.1212 × 10^−7^ mol·cm^−3^

In Equation (A4):

$$kr_{{CO}_{2}}=\frac{slope[10\%\;{CO}_{2}]}{C_{A10\%{CO}_{2}}}$$

$${kr}_{{CO}_{2}}=\frac{0.0114\;{cm}^{3}}{7.1212\times 10^{-7}\times 3600\;mols}$$

$${kr}_{{CO}_{2}}=4.44\;{cm}^{3}{mol}^{-1}s^{-1}$$

Using these constants and Equation (7), the rate of carbon gasification (χ) can be calculated for pilot testing the physical activation of palm shells carbonized in the Nichols furnace at 960 °C. The results are presented in Table A2.

**Table A2:** Gasification rate of carbon (χ) for the pilot test of physical activation of carbonized palm K shells in the Nichols furnace at 960 °C with an atmosphere of 10% CO_2_-26% H_2_O.

Time (h)	Gasification Rate of Carbon—χ (%)	
	Experimental	Predicted
0	-	0.00
1	-	8.48
2	10.00	16.32
3	18.50	23.56
4	26.10	30.26
5	33.50	36.45
6	43.00	42.18
7	51.00	47.48
8	56.00	52.39
9	60.69	56.94
10	65.00	61.16
11	69.00	65.07
12	75.00	68.70
13	85.00	72.07
14	95.00	75.20
15	98.00	78.12

## References

[1] Ello A.S., de Souza L.K.C., Trokourey A., Jaroniec M. (2013). Coconut shell-based microporous carbons for CO_2_ capture. Microporous Mesoporous Mater..

[2] Hidayu A.R., Muda N. (2016). Preparation and Characterization of Impregnated Activated Carbon from Palm Kernel Shell and Coconut Shell for CO_2_ Capture. Procedia Eng..

[3] Rashidi N.A., Yusup S. (2017). Potential of palm kernel shell as activated carbon precursors through single stage activation technique for carbon dioxide adsorption. J. Clean. Prod..

[4] Shoaib M., Al-Swaidan H.M. (2015). Optimization and characterization of sliced activated carbon prepared from date palm tree fronds by physical activation. Biomass Bioenergy.

[5] Xia C., Shi S.Q. (2016). Self-activation for activated carbon from biomass: Theory and parameters. Green Chem..

[6] de la Torre E., Gámez S. (2019). Not Reacted Core Model Applied in Palm Nut Shell Pyrolysis. Int. J. Chem. Eng..

[7] Gómez A., Klose W., Rincón S. (2008). Pirólisis de Biomasa.

[8] Gómez A., Klose W., Rincón S.Y. (2010). Carbón Activado de Cuesco de Palma: Estudio de Termogravimetría y Estructura.

[9] Raveendran K., Ganesh A., Khilar K.C. (1995). Influence of mineral matter on biomass pyrolysis characteristics. Fuel.

[10] Sun S., Yu Q., Li M., Zhao H., Wu C. (2019). Preparation of coffee-shell activated carbon and its application for water vapor adsorption. Renew. Energy.

[11] Pallarés J., González-Cencerrado A., Arauzo I. (2018). Production and characterization of activated carbon from barley straw by physical activation with carbon dioxide and steam. Biomass Bioenergy.

[12] Daud W., Ali W. (2004). Comparison on pore development of activated carbon produced from palm shell and coconut shell. Bioresour. Technol..

[13] Manocha S.M. (2003). Porous carbons. Sadhana.

[14] Marsh H., Reinoso F.R. (2006). Activated Carbon.

[15] Shabir S., Hussain S.Z., Bhat T.A., Amin T., Beigh M., Nabi S. (2024). High carbon content microporous activated carbon from thin walnut shells: Optimization, physic-chemical analysis and structural profiling. Process Saf. Environ. Prot..

[16] Tsai W.-T., Jiang T.-J. (2018). Mesoporous activated carbon produced from coconut shell using a single-step physical activation process. Biomass Convers. Biorefinery.

[17] Vi N.N.T., Truyen D.H., Trung B.C., An N.T., Van Dung N., Long N.Q. (2017). Porous carbon from local coconut shell char by CO_2_ and H_2_O activation in the presence of K_2_CO_3_. AIP Conference Proceedings.

[18] McDougall G.J., Hancock R.D. (1981). Gold complexes and activated carbon. Gold Bull..

[19] Prauchner M.J., Rodríguez-Reinoso F. (2012). Chemical versus physical activation of coconut shell: A comparative study. Microporous Mesoporous Mater..

[20] Jiang C., Yakaboylu G.A., Yumak T., Zondlo J.W., Sabolsky E.M., Wang J. (2020). Activated carbons prepared by indirect and direct CO_2_ activation of lignocellulosic biomass for supercapacitor electrodes. Renew. Energy.

[21] Rodríguez-Reinoso F., Molina-Sabio M., González M.T. (1995). The use of steam and CO_2_ as activating agents in the preparation of activated carbons. Carbon.

[22] Kunii D., Chisaki T. (2007). Rotary Reactor Engineering.

[23] Rambabu N., Rao B.V.S.K., Surisetty V.R., Das U., Dalai A.K. (2015). Production, characterization, and evaluation of activated carbons from de-oiled canola meal for environmental applications. Ind. Crops Prod..

[24] Heidarinejad Z., Dehghani M., Heidari M., Javedan G., Ali I., Sillanpää M. (2020). Methods for preparation and activation of activated carbon: A review. Environ. Chem. Lett..

[25] Jabbar A. (2020). Comparative study for adsorption of acidic and basic dyes on activated carbon prepared from date stone by different activation agent. Al-Qadisiyah J. Eng. Sci..

[26] Niu J., Shen Y., Zhang H., Li L., Guo S. (2024). Preparation of highly microporous activated carbon by utilizing inherent iron in coal through CO_2_ and steam co-activation for improving CO_2_ capture and methylene blue removal. Fuel.

[27] McDougall G.J. (1991). The physical nature and manufacture of activated carbon. J. S. Afr. Inst. Min. Metall..

[28] Cagnon B., Py X., Guillot A., Stoeckli F., Chambat G. (2009). Contributions of hemicellulose, cellulose and lignin to the mass and the porous properties of chars and steam activated carbons from various lignocellulosic precursors. Bioresour. Technol..

[29] Oudenne P. (1990). La experiencia en la carbonización, activación y regeneración de carbón activado. Simposio Metalurgia Extractiva.

[30] (1986). Standard Test Method for Determination of Iodine Number of Activated Carbon.

[31] (1978). Standard for Powdered Activated Carbon.

[32] Green D.W., Southard M.Z. (2018). Perry’s Chemical Engineers’ Handbook.

